# Motivation-Enhancing Psychotherapy for Inpatients With Anorexia Nervosa (MANNA): A Randomized Controlled Pilot Study

**DOI:** 10.3389/fpsyt.2021.632660

**Published:** 2021-02-01

**Authors:** Katrin Ziser, Nadine Rheindorf, Katharina Keifenheim, Sandra Becker, Gaby Resmark, Katrin E. Giel, Eva-Maria Skoda, Martin Teufel, Stephan Zipfel, Florian Junne

**Affiliations:** ^1^Department of Psychosomatic Medicine and Psychotherapy, University Hospital Tuebingen, Tuebingen, Germany; ^2^Clinic for Psychosomatic Medicine and Psychotherapy, LVR-University Hospital, University Duisburg-Essen, Essen, Germany; ^3^Centre of Excellence for Eating Disorders, University of Tuebingen, Tuebingen, Germany

**Keywords:** anorexia nervosa, inpatient treatment, psychotherapy, readiness to change, ambivalence, therapeutic alliance

## Abstract

Patients with anorexia nervosa (AN) are frequently characterized by an unstable readiness to change and high ambivalence toward treatment. Enhancing readiness to behavioral change therefore plays an essential role for adherence to treatment especially for severely ill patients treated in inpatient settings. Therefore, a novel 10 week program for the individual psychotherapy sessions was designed using elements from motivational interviewing to be applied within the multidisciplinary inpatient treatment for patients with AN. In a randomized controlled pilot trial, *N* = 22 patients with AN received either the new intervention or treatment as usual in one of two recruiting university hospitals. Readiness to change, eating disorder pathology, therapeutic alliance as well as acceptance and feasibility of the new intervention were measured from patients and therapists in week 1, 5, and 10 of inpatient treatment. Results confirm acceptance and feasibility of the MANNA intervention as evaluated by patients as well as therapists. Patients receiving the new intervention completed their inpatient treatment significantly more often on regular terms than patients receiving treatment as usual. No differences between the groups could be found concerning therapeutic alliance during and at the end of treatment and readiness to change. Absolute numbers of BMI increase indicate a larger increase in the intervention group albeit not significant in this pilot study sample. Limitations of the study such as the small sample size as well as possible adaptions and advancements of the intervention that need to be examined in a larger clinical trial of efficacy are discussed. This phase II study is registered with the German Clinical Trials Register (DRKS) under the trial number DRKS00015639.

## Introduction

Anorexia nervosa (AN) is an eating disorder characterized by significantly low body weight, an intense fear of weight gain or becoming fat and body image disturbances [DSM-5; (1)]. Due to their low weight, patients are at risk for somatic complications such as cardiovascular complications, impairment of the gastro-intestinal tract or osteoporosis (2). Psychological sequelae such as depressed mood, social isolation, and a low quality of life are also highly prevalent (2–4). Taken all of these risks together, AN is known to be the mental disorder with the highest mortality rate in young females (5). Despite the seriousness of the disorder, patients with AN frequently experience an unstable readiness to change and high ambivalence toward treatment due to the ego-syntonic nature of the eating disorder (6).

This is especially relevant for severely ill patients who need high intensity treatment in inpatient settings due to potentially life-threatening stages of the disease. Patients with severe AN are usually admitted to inpatient therapy under difficult conditions such as very low body weight with acute malnutrition including e.g., disturbed serum minerals and the risk of refeeding-syndrome. They might have experienced failure of multiple other treatment approaches in varying settings in the past and may experience emotional pressure from families/friends to seek therapy. Still, patients with AN often display insufficient comprehension of the severity of their medical situation and might even oppose weight gain. The therapeutic structures however usually include contingency contracts for controlled weight gain as one of the main goals of treatment. They often foster ambivalence since patients with AN often may want to “overcome” the eating disorder but are reluctant to any weight gain (7, 8).

In Germany, inpatient treatment for patients with AN is advised if the body mass index (BMI) is below 15 kg/m^2^, rapid weight decrease happened (<20% in 6 months) or if there are severe other eating disorder symptoms, psychological, familial, or social factors that make success in outpatient or partial hospitalization settings unlikely (9). In addition to somatic monitoring and treatment, patients with AN routinely receive high doses of individual psychotherapy (2–3 sessions per week) as well as group psychotherapy, nutritional counseling, body-oriented therapy, and art or music therapy. The inpatient treatment is usually provided by a multidisciplinary treatment team consisting of physicians, psychologists, specialized nurses, nutritionists, physiotherapists, and other specialized therapists.

Despite efforts to improve outcomes through the described multidisciplinary treatment settings, about one third of patients did not show a significant response to inpatient therapy in a study by Schlegl et al. (10). Their results from analyzing a sample of over 400 patients with AN however emphasize the relevance of high internal motivation as a predictor of good outcome in therapy at the time of discharge (10). They therefore suggest the utilization of techniques for enhancing motivation and increasing patients' readiness to change such as motivational interviewing (MI).

MI according to Miller and Rollnick (11) is an approach for helping people to change their thinking and behavior. It was initially developed for the areas of substance abuse and health-related problems (e.g., smoking cessation) and has since been applied and shown promise for behavior change in various medical care settings such as dangerous drinking, dental caries, smoking abstinence, quality of life, and self-monitoring as well as psychotherapy (12, 13). Especially in psychotherapy, MI is frequently not used as a “stand-alone treatment” but integrated as the framework or stance under which psychotherapeutic interventions are conducted (14).

Due to the primary goal of resolving ambivalence and increasing intrinsic motivation to change, integrating MI into the treatment of patients with AN seems promising (15, 16). A review found mixed results but some promise of MI interventions in the field of eating disorders (17). For the treatment of AN specifically, more recent studies also show support for MI in enhancing treatment adherence and building a good therapeutic alliance between patients and therapists (18, 19).

Considering these aspects, we aimed at developing an intervention for the inpatient treatment of severely ill patients with AN. This intervention integrates MI into established inpatient intervention modules/techniques in the treatment of AN to enable incremental adaptation of treatment settings in case of the success of the trial. The aim is to increase intrinsic motivation to change in patients with AN, improve adherence to treatment (reduce dropouts) and strengthen therapeutic alliance.

For the development of the novel treatment manual, structural guidance was taken from Carroll and Nuro (20) with regard to developing a stage I psychotherapy manual suitable for pilot and feasibility testing. The authors provide advice on the general outline of such manuals in terms of elements to be included [see Table 2 in (20)], elements critical to this stage of development and suggest a model for delineating treatments. This guided approach enables the development of a “clinician-friendly” manual that can facilitate its implementation into clinical practice.

The objective of the present pilot study was to investigate the newly developed manual for inpatient treatment of patients with AN in comparison to the usual treatment concerning its acceptance and feasibility as well as impact on treatment adherence and therapeutic alliance. Our hypotheses were: (1) The investigated intervention is acceptable and feasible for patients as well as therapists, (2) Patients receiving the new intervention show a higher motivation to change, better treatment adherence as well as a stronger therapeutic alliance than patients receiving the treatment-as-usual, (3) Patients receiving the new intervention show greater weight gain and improvement in eating disorder associated psychopathology than patients receiving the treatment-as-usual (exploratory analysis). Additionally, one important aim of this study was to gain insight into (subjectively) needed improvements and adaptions to the intervention as suggested by patients and therapists.

## Materials and Methods

### The MANNA Intervention

The therapeutic style used in MANNA is that of MI according to Miller and Rollnick (11) as described in the introduction. Two experienced experts performed training sessions with all study therapists on the theoretical background and structure of MI, different techniques as well as conducting practice exercises. Emphasis in the training was put on the therapeutic stance of MI as well as basic skills.

Basic skills in MI are represented by the OARS acronym. O thereby refers to asking open questions, A refers to using affirmations, R to reflective listening and S to summarizing. Additionally giving information and advice is a basic skill used to prevent the therapist from adopting the role of “the expert” and providing uninvited advice to the patient (e.g., “You really should quit…,” “I would...”) (14).

**Figure 1:**
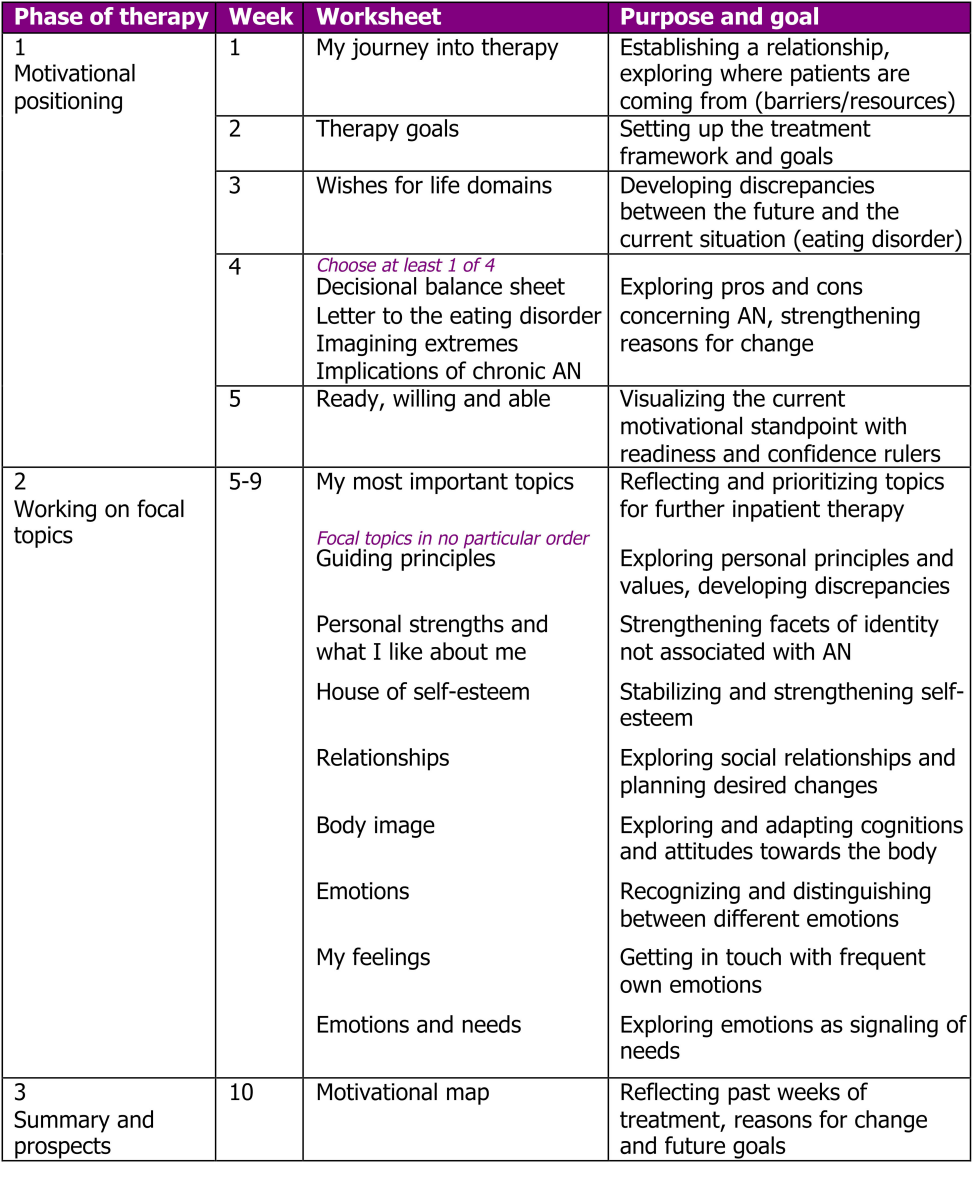
Overview of the MANNA intervention.

Core of the intervention materials and orientation over the course of treatment are worksheets that are worked on (often after the patient has started the worksheet on his/her own as a homework) at a mean frequency of one worksheet per week. This mean frequency was chosen to account for the structured environment of inpatient setting on one hand and allow for the need for flexibility to address the patients' individual needs and potential comorbidities by the therapist on the other hand. An overview and brief descriptions of the worksheets of the MANNA intervention can be found in Figure 1.

The MANNA intervention was designed for the first 10 weeks of inpatient individual psychotherapy sessions in the multidisciplinary treatment of severely ill patients with AN. The manual is based on the principles of motivational interviewing and the related therapeutic techniques and it contains elements of the Maudsley Model of Anorexia Nervosa Treatment for Adults [MANTRA; (21)]. Furthermore, the manual includes interventions that have been successfully integrated in in the treatment of eating disorders before (such as letter to the eating disorder as a friend/foe, or explicit therapy goals for the time of inpatient treatment).

Each worksheet exists in a patient version that is to be distributed to the patient as well as a therapist version. The therapist version consists of three sections: (1) a summary of purpose and goals of the worksheet, (2) instructions/reflections for the use of motivational interviewing for discussing this particular worksheet with the patient, and (3) helpful phrases for the therapist, considerations for different motivational stages or potential “therapeutic traps.” This version also functions as a summary of the most important aspects of the present worksheet that can help the therapist to orientate himself/herself within the MANNA intervention on quick glance before a therapy session in the frequently time-limited inpatient setting.

The course of individual therapy on the MANNA intervention is divided into three phases. Phase 1 (weeks 1–4) is about getting to know the patient and building a therapeutic relationship and the working alliance, exploring reasons for undergoing inpatient therapy, exploring short- as well as long-term goals of the patients and identifying how the AN disorder seems to help or where it hinders to achieve these life-goals of the patient. Biographical aspects and other factors that contributed to the development of AN are discussed. All provided worksheets in this phase are obligatory.

To provide some flexibility to therapists, week 4 contains a selection of four alternative worksheets all addressing pros and cons of AN and beliefs associated with AN in different ways. This enables the therapist to choose the worksheet that seems most suitable to the patient at the present moment (e.g., choosing a narrative task vs. a cognitive-rationale task). Additionally, the alternative worksheets can further be used in case ambivalence remains very high at this stage and needs further exploring and developing discrepancies between the status quo and wishes/goals for the future.

In transition to phase 2 (starting in week 5), readiness and confidence rulers are used to visualize the motivational standpoint of the patient and planning steps for the further course of treatment. This involves the selection and prioritization of focal treatment topics by the patient together with her therapist. A variety of potential topics are given on worksheet 6, representing topics patients with AN frequently struggle with including self-esteem (22), identity (23), relationships/social interactions (24), body image (25) as well as emotions, and needs (26). Patients can add own topics and therapists can bring in own worksheets accordingly as long as they are discussed in the therapeutic style of motivational interviewing.

Phase 3, the end of the MANNA therapy, entails a central worksheet called “motivational map” on which significant parts of the last 10 weeks of treatment are integrated: Patients principles and values, long-term goals in different areas of the patients life, consequences of the eating disorder and what motivates the patients to move forward with regard to their recovery from AN as well as the next goals in treatment and focal topics for further (outpatient) treatment. This enables a reflection of the past individual therapy sessions, visualizes the patients current motivational stance, and can be a summary that facilitates transition into a setting of partial hospitalization or outpatient care.

### Sample

Patients with AN being admitted to one of the two university hospital study sites for specialized inpatient treatment were invited to participate in this study over a period of 1 year. Inclusion criteria consisted of a minimum age of 18 years and full-syndrome AN according to the DSM-5. Exclusion criteria were a BMI below 12 kg/m^2^ since continuous attendance of individual psychotherapy sessions cannot be guaranteed below that weight due to probable cognitive impairments or somatic complications. Further exclusion criteria were: comorbidities of schizophrenia spectrum disorders, bipolar disorder as well as current substance abuse. Notably, although being female was not an inclusion criterion, only female patients with full-syndrome AN presented at the recruiting sites during the study period.

### Measures

The following measures and questionnaires were presented to all participants.

#### Diagnostic and Clinical Interviews (SCID-I, EDE Interview)

The German version of the Structured Clinical Interview for DSM-IV [SCID-I; (27)] was administered and adopted to fit DSM-5 criteria (the SCID interview for DSM-5 was not yet available in German). It is a semistructured interview guide for administering valid diagnoses according to DSM-5 and was used to assess comorbidities in the present sample. For verification of the AN diagnosis and exploration of eating disorder pathology, the Eating Disorder Examination Interview [EDE-I; (28)] was administered.

#### Sociodemographic and Closure Questionnaires

At the beginning of the diagnostic interview, patients filled in a demographic questionnaire with basic information such as gender, age, living situation, education as well as year of initial diagnosis of AN and former treatments (if any). Height and weight were measured in the inpatient unit at admission (and regularly during the course of treatment) and were extracted from the patients' clinical file.

At the end of inpatient treatment or at the end of the study period (week 10), therapists filled in a closure questionnaire for each participating patient that documented the date of discharge from inpatient treatment and kind of discharge (e.g., regular treatment termination, dropout of treatment, need to transfer the patient to another department or another hospital) as well as other characteristics of the treatment course such as changes of therapists.

#### Psychiatric Status Rating (PSR) for AN

The German version of the Psychiatric Status Rating [PSR; (29)] is a rating completed by the therapist to evaluate the patient's current psychopathological state and indicates the severity of the disorder (i.e., AN). It consists of 6 stages ranging from 1 (no symptoms of AN) to 6 (severe symptoms of AN) whereof the ratings 5 and 6 refer to full-syndrome AN according to the DMS-5.

#### University of Rhode Island Change Assessment—Short (URICA-S)

The University of Rhode Island Change Assessment—Short [URICA-S; (30)] is a self-report measure for assessing the four stages of change according to the transtheoretical model [TTM; (31)]. A total of 16 items are rated on a 5-point likert scale from 0 (do not agree at all) to 4 (agree very strongly) which can computed into the four subscales precontemplation, contemplation, action, and maintenance. Internal consistencies in the present sample proved to be good with Cronbachs α between 0.562 and 0.850 for the contemplation, action and maintenance scales. A floor effect for the precontemplation scale could be observed which was however to be expected. Since all of the patients decided to attend inpatient therapy for their AN, precontemplation was expected to be very low. Otherwise the decision for receiving treatment would likely not have been made by the patients.

#### Helping Alliance Questionnaire (HAQ)

The Helping Alliance Questionnaire (32) is an instrument assessing the therapeutic alliance in therapy. 11 items are rated on a 6-point likert scale (0 not at all −5 very much) that are computed to the two subscales “relation to the therapist” and “satisfaction with therapeutic outcome” which can be combined to a total score of therapeutic alliance. The HAQ can be used in a self-report version (e.g., patients' perspective) as well as a third-party assessment (e.g., rated by the therapist). Both versions were used in the present study and proved to be reliable measures with Cronbachs α = 0.585 −0.919.

#### Eating Disorder Pathology (EDE-Q)

The Eating Disorder Examination—Questionnaire (33) is the questionnaire version of the Eating Disorder Examination Interview used for diagnostics and was used as an indication of eating disorder pathology in the course of treatment. Twenty-four items are computed to the four subscales “restraint,” “eating concern,” “weight concern,” and “shape concern.” Internal consistencies of the EDE-Q proved to be excellent in the present study with Cronbachs α = 0.730 −0.989.

#### Acceptance and Feasibility Questionnaire

A self-administered questionnaire was used for assessing acceptance, feasibility and benefits from the patients' perspective on a 5-point likert scale as well as free text for comments and suggestions for improvement. Therapists that had patients in the intervention group also gave feedback on acceptance and feasibility of the MANNA intervention as well as a rating of benefits and possible improvements of the individual worksheets of the MANNA treatment.

### Procedure

Patients were informed about the study and invited to participate consecutively upon presentation for inpatient treatment at one of the two participating university hospitals. If they consented to participate, patients were randomly assigned to the intervention, or the control group according to predefined randomization lists. The patients' individual psychotherapist was informed about the inclusion of the patient into the study and her allocation to the intervention or control group. An appointment for the diagnostic interview with an independent interviewer (not the individual therapist) was scheduled before or within the first days of inpatient treatment. Patients underwent the diagnostic interview and received the MANNA treatment in individual psychotherapy sessions (intervention group) or the treatment-as-usual (control group). Questionnaires were filled in in week 1, 5, and 10 of inpatient treatment by the patient as well as the individual psychotherapist. For all patients, (regular or irregular) end of treatment as well as changes in psychotherapists and other events were documented.

This study was carried out in accordance with the recommendations of good clinical practice. The protocol was approved by the ethics committee of the medical faculty of the University of Tuebingen (No. 148/2018BO1) as well as the ethics committee of the medical faculty of the University of Duisburg-Essen (No. 19-8653-BO). All participants gave written informed consent in accordance with the Declaration of Helsinki. The study was registered with the German Clinical Trials Register (DRKS) under the trial number DRKS00015639.

### Analyses

All statistical analyses were performed in IBM SPSS Statistics (version 27). The level of significance for all analyses was set at α = 0.05. Means, standard deviations and percentages are reported for sample descriptions. Kolmogorov-Smirnov tests were used to assess variables for normal distribution. *T*-tests were used for normally distributed variables and Mann-Whitney-*U*-tests for not normally distributed variables to assess differences between the two study groups at baseline and at the end of treatment. For all single comparisons, Cohens *d* is reported as a measure of effect sizes. According to Cohen (34), *d* > 0.2 thereby indicates a small effect, *d* > 0.5 a medium effect and *d* > 0.8 a large effect. For the comparison of the distributions of AN subtypes in the two study groups at baseline, a chi-squared test is used. To assess treatment adherence, Fishers exact test and the subsequent calculation of an odds ration including a confidence interval are reported.

## Results

A total of 27 patients initially agreed to participate in the study. After omitting data sets of patients that did not hold up with their diagnosis of full syndrome AN during the diagnostic interview or were scheduled but not admitted to inpatient therapy, a total of *N* = 22 females participated in the study.

### Descriptive Statistics

An overview of the demographic and clinical characteristics of the participants at baseline can be found in Table 1.

**Table 1:** Demographic and clinical characteristics of the study population at baseline (*N* = 22).

	**Intervention group (*n* = 11)**	**Control group (*n* = 11)**	
**Variable**	***M (SD)***	***M (SD)***	**Analysis**
Age	31.5 (9.5)	31.9 (12.6)	*U* = 56.50, *p* = 0.797, *d* = 0.11
BMI	15.6 (1.3)	15.3 (1.5)	*t* (19) = −0.51, *p* = 0.614, *d* = −0.22
Illness duration in years	10.9 (8.6)	7.2 (5.9)	*t* (17) = −1.09, *p* = 0.290, *d* = −0.50
No. of comorbidities	1.1 (1.0)	2.0 (1.9)	*U* = 38.00, *p* = 0.393, *d* = 0.41
AN subtype			*χ^2^*(1) = 0.19, *p* =0.665
- restrictive - binge-purge	45.5 % 54.5 %	36.4 % 63.6 %	
**EDE-Q**			
- restraint - eating concern - weight concern - shape concern - sum score	4.4 (2.0) 3.4 (2.0) 4.1 (1.6) 4.4 (1.6) 4.1 (1.7)	4.6 (1.6) 3.7 (1.5) 4.5 (1.4) 5.0 (0.8) 4.5 (1.2)	*U* = 59.50, *p* = 0.949, *d* = 0.03 *t* (20) = 0.51, *p* = 0.617, *d* = 0.22 *t* (20) = 0.66, *p* = 0.519, *d* = 0.28 *U* = 51.50, *p* = 0.562, *d* = 0.25 *U* = 56.00, *p* = 0.797, *d* = 0.13
PSR	5.2 (0.6)	5.6 (0.5)	*U* = 35.00, *p* =0.173, *d* = 0.65
**URICA-S**		
- precontemplation - contemplation - action - maintenance	0.6 (0.5) 3.2 (0.5) 3.3 (0.6) 2.9 (1.0)	0.4 (0.4) 3.2 (0.9) 2.7 (1.0) 2.0 (1.4)	*U* = 41.50, *p* = 0.217, *d* = 0.55 *t* (20) = 0.15, *p* = 0.884, *d* = 0.06 *t* (20) = −1.68, *p* = 0.109, *d* = −0.72 *t* (20) = −1.80, *p* = 0.088, *d* = −0.77
**HAQ sum score**			
- self-report - therapist report	44.3 (9.1) 33.5 (5.2)	35.3 (7.0) 33.8 (7.2)	*t* (19) = −2.56, ***p*** **=** **0.019**, *d* = −1.12 *t* (18) = 0.11, *p* = 0.916, *d* = 0.05

*AN, Anorexia nervosa; BMI, body mass index (kg/m^2^); EDE-Q, Eating Disorder Examination-Questionnaire; HAQ, Helping Alliance Questionnaire; PSR, Psychiatric Status Rating; URICA-S, University of Rhode Island Change Assessment – Short*.

Unfortunately, there was a significant difference at baseline concerning the HAQ sum score in the self-report version between the intervention and the control group with patients in the intervention group rating the therapeutic alliance to be better (indicated by a higher score) than patients in the control group. There were no other significant differences of both groups at baseline.

### Acceptance and Feasibility

Overall acceptance and feasibility of the MANNA intervention was rated high to very high by patients as well as therapists concerning nearly all investigated aspects. The individual ratings can be found in Table 2.

**Table 2:** Evaluation of acceptance and feasibility of the MANNA intervention.

	**Patients (*n* = 9)**	**Therapists (*n* = 9)**
**Evaluation**	***M (SD)***	***M (SD)***
Personal/Patients overall benefits from the MANNA intervention	3.3 (1.1)	3.6 (0.7)
Overall satisfaction with the worksheets	4.7 (0.7)	4.2 (0.8)
Comprehensibility of the worksheets	4.6 (0.7)	4.4 (0.5)
Usefulness of the worksheets	3.7 (1.4)	3.9 (0.9)
Logical sequencing of worksheets	3.8 (0.8)	4.1 (0.6)
Balance of worksheets and space for emerging topics	3.9 (1.1)	3.9 (1.1)
Essential topics in the first weeks of treatment were missing	2.2 (1.2)	1.6 (0.9)
Usefulness of therapists instruction sheet		4.3 (0.5)

*Ratings were given on a 5-point likert scale (1–5) with higher values indicating higher acceptance or satisfaction*.

*Bold values indicate significant results*.

Concerning the question of “missing topics in the first weeks of treatment,” the low average ratings in this regard indicated that patients were satisfied with the topics addressed in phase 1 and no essential topics were missing in the MANNA intervention from a patients perspective. As for comments on potential useful additions to the intervention, patients indications were mainly related to worksheet 2 (goals for inpatient therapy) for which one patient wished to shorten this process of writing down inpatient therapy goals and defining steps toward achieving them. Whereas, another patient wished to discuss inpatient therapy goals in more detail and would like to add a more creative approach (such as visual or narrative accounts). Another useful addition might be a designated worksheet to explore more about the family background, as suggested by a patient as well as a therapist. Finally, compiling the worksheets in a therapy folder and/or incorporating accompanying tasks such as small homework or therapy diary task was suggested by a patient.

### Readiness to Change, Treatment Adherence, and Therapeutic Alliance

Concerning treatment adherence, patients of the intervention group completed inpatient treatment on regular terms significantly more often than patients of the control group who dropped out or were transferred or discharged before the intended end of treatment more often. The odds ratio indicated that patients of the control group were nearly eight times more likely to drop out of treatment although the confidence interval indicates a very large possible range.

There were no significant differences concerning readiness to change as measured in the URICA-S at the different measurement time points between the intervention and the control group in this pilot sample of patients. For a detailed account of the single comparisons, see Table 3.

**Table 3:** Single comparisons between the study groups at week 5 (t1) and at the end of treatment (t2).

	**Intervention group** *n* = 11*	**Control group** *n* = 11*	
**Variable**	***M (SD)***	***M (SD)***	**Analysis**
**READINESS TO CHANGE, TREATMENT ADHERENCE AND THERAPEUTIC ALLIANCE**			
**URICA-S t1**				
- precontemplation - contemplation - action - maintenance	0.3 (0.4) 3.1 (0.7) 3.3 (0.5) 2.4 (1.3)	0.2 (0.2) 3.4 (0.6) 3.2 (0.7) 2.5 (0.9)	*U* = 34.00, *p* = 0.633, *d* = 0.25 *t* (16) = 1.06, *p* = 0.307, *d* = 0.50 *t* (16) = −0.23, *p* = 0.818, *d* = −0.11 *t* (16) = 0.18, *p* = 0.861, *d* = 0.08
**URICA-S t2**			
- precontemplation - contemplation - action - maintenance	0.2 (0.3) 2.9 (0.6) 3.4 (0.5) 2.3 (0.6)	0.4 (0.4) 3.2 (0.9) 3.3 (0.8) 2.6 (0.9)	*t* (7) = 1.10, *p* = 0.307, *d* = 0.74 *t* (7) = 0.61, *p* = 0.563, *d* = 0.41 *t* (4.5) = −0.20, *p* = 0.854, *d* = −0.14 *t* (7) = 0.51, *p* = 0.625, *d* = 0.34
Irregular treatment termination^+^	2	7	Fishers exact test ***p*** **=** **0.040**, OR = 7.88, CI [1.11; 56.12]
**HAQ sum score t1**			
- self-report - therapist report **HAQ sum score t2** - self-report - therapist report	45.0 (5.6) 35.9 (9.0) 45.5 (4.2) 37.5 (3.5)	40.5 (8.7) 35.6 (7.3) 43.8 (5.6) 36.4 (6.3)	*t* (16) = −1.33, *p* = 0.202, *d* = −0.63 *U* = 34.00, *p* = 0.633, *d* = 0.25 *t* (6) = −0.50, *p* = 0.633, *d* = −0.36 *t* (7) = −0.31, *p* = 0.766, *d* = −0.21
**WEIGHT GAIN AND PSYCHOPATHOLOGY AT THE END OF INPATIENT TREATMENT (EXPLORATORY ANALYSES)**			
BMI **t2** BMI gain **t0 to t2**	16.6 (1.0) 1.79 (0.9)	16.5 (1.5) 1.26 (0.8)	*t* (7) = −0.12, *p* = 0.906, *d* = −0.08 *t* (7) = −0.95, *p* = 0.375, *d* = −0.64
**EDE-Q t2**			
- restraint - eating concern - weight concern - shape concern - sum score	1.3 (0.9) 1.6 (1.3) 2.2 (1.0) 2.9 (1.5) 2.0 (1.0)	2.5 (2.4) 2.8 (2.0) 2.8 (1.9) 3.9 (1.6) 3.0 (1.0)	*t* (3.7) = 0.90, *p* = 0.425, *d* = 0.66 *t* (7) = 1.11, *p* = 0.304, *d* = 0.74 *t* (7) = 0.61, *p* = 0.559, *d* = 0.41 *t* (7) = 0.98, *p* =0.360, *d* = 0.66 *t* (7) = 1.00, *p* = 0.352, *d* = 0.67
PSR **t2**	4.8 (0.4)	4.6 (1.1)	*U* = 11.50, *p* = 0.841, *d* = 0.13

BMI, body mass index (kg/m^2^); CI, confidence interval; EDE-Q, Eating Disorder Examination-Questionnaire; HAQ, Helping Alliance Questionnaire; OR, Odds ratio; PSR, Psychiatric Status Rating; t1, week 5 of treatment; t2, week 10 of treatment; URICA-S, University of Rhode Island Change Assessment—Short; *n refers to the sample size at baseline (week 1),

+ *irregular treatment terminations consisted of dropouts, transfers to another clinic/department or termination by the treatment team. Bold values indicate significant results*.

Contrary to our hypothesis, no significant differences of the therapeutic alliance ratings in self-report as well as therapist report could be found between the intervention group and the control group for the different measurement time points except for the difference between groups in the self-report version at baseline. Potential reasons for this difference are examined in the discussion section.

### Exploratory Analyses of Weight Gain and Psychopathology

Exploratory completer analyses were performed for differences in increase in BMI and decrease in eating disorder psychopathology at the end of the MANNA intervention. Mean BMI increase in the intervention group from baseline to the end of the MANNA intervention was higher than in the control group (1.79 vs. 1.26). This indicates a larger BMI increase in the intervention group albeit not significant in this small pilot study sample. Details of these comparisons are also reported in Table 3.

Concerning eating disorder psychopathology, absolute numbers indicate a lower EDE-Q sum score in the intervention group than the control group at the end of inpatient treatment but no significant differences between emerged concerning any scales of the EDE-Q. The PSR as rated by the respective therapists also indicated no differences between the intervention and the control group at the end of treatment.

## Discussion

This pilot study of the novel MANNA intervention for inpatients with AN examined acceptance, feasibility and outcomes in German inpatient settings as well as its effects on treatment adherence and therapeutic alliance compared to treatment as usual. The MANNA intervention thereby proved to be very well-accepted and feasible according to its evaluations by patients as well as therapists, thus confirming the first hypothesis.

Beyond its acceptance and feasibility, patients receiving the MANNA intervention completed their treatment significantly more often on regular terms compared to patients receiving treatment as usual that terminated treatment irregularly more often (through dropout, transfer to another clinic/department or termination by the treatment team). This indicates a higher treatment adherence of patients in the intervention group and confirms our second hypothesis about the positive influence of the MANNA intervention on treatment adherence. Since treatment dropout can be seen as one of the major risks in early stages of therapy for inpatients with AN (35), this effect can be seen as a very promising finding toward the potential effects of the novel approach of the MANNA intervention.

The other part of our second hypothesis however, concerning the MANNA intervention improving therapeutic alliance could not be confirmed in this pilot sample. There were no differences of patients' perception of the therapeutic alliance with their individual psychotherapist during or at the end of inpatient treatment between the intervention and the control group. At baseline, patients in the intervention group indicated a stronger subjective therapeutic alliance than patients in the control group. This difference could originate in several reasons.

On one hand, the small sample size could have produced this difference in therapeutic alliance ratings by chance. The effect would subsequently diminish in a larger sample size. As another possibility, a bias by therapist could have the difference although we tried to control for therapists influence on treatment effects by randomizing patients. Therefore, participating therapists had patients in the intervention group as well as patients in the control group which makes the assumption of a therapist effect less likely.

On the other hand, the difference in therapeutic alliance ratings at baseline might have been an early product of the MANNA intervention. Since the baseline questionnaires were frequently given out by the individual therapist, patients might have already had some interactions with their individual therapists (e.g., admission session). They therefore came in contact with the MANNA intervention and the therapeutic stance of motivational interviewing, possibly resulting in the initiation of a stronger early therapeutic alliance. This effect might have dissolved over the course of treatment when patients in the control group got to know their individual therapists better and rated their respective therapeutic alliance comparable to the intervention group.

In the literature, we could not find valid evidence for the impact of MI on early therapeutic alliance, therefore neither supporting nor weakening this assumption. There was one therapists' report about building up a good therapeutic relationship in a MI-based treatment of patients with AN from the MOSAIC trial (18). This process evaluation did however not specifically address early therapeutic alliance.

Apart from the direct effect of MI on early therapeutic alliance, evidence could be found for the effect of motivation to change. A study with inpatients with AN by Marzola et al. (36) shows the importance of motivation to change as a prerequisite or a moderator of early clinical improvement and the formation of a strong therapeutic alliance. Since strengthening motivation to change is one of the key goals and effects of MI, this finding might also apply to MI.

Assuming therapists utilization of MI in the treatment of inpatients with AN strengthens early therapeutic alliance implies other possible effects: A cohort study about adolescent patients with AN showed that a higher rating of early therapeutic alliance was associated with reaching the target weight faster irrespective of the treatment setting (37). Another study with adult outpatients with AN however showed no impact of early therapeutic alliance on changes in weight but in parts of eating disorder pathology (namely restraint and shape concern) (38). Although at this time these are speculative assumptions that should be examined in future studies, the utilization of MI such as in the MANNA intervention might strengthen early therapeutic alliances which positively affect the outcome of treatment and/or changes in eating disorder pathology.

Only partial support could be found in the present pilot study for the third exploratory hypothesis. Patients in the intervention group did show greater BMI increase and improvement in eating disorder associated psychopathology at the end of inpatient treatment compared to the control group in absolute numbers. However, these differences did not turn out to be statistically significant. This probably originates in the small sample size at this measurement time point (four and five patients, respectively), therefore a larger sample could examine the validity of these differences.

Concerning further development of the treatment manual of the MANNA intervention, there are some advances that can be made for a stage II manual for a phase III MANNA study. According to Carroll and Nuro (20) these may lay in the explication of procedures and standards for therapist selection, in further elaborating the training and supervision of therapists conducting the intervention as well as in implementing guidelines for troubleshooting. From the experience with therapist trainings in the current study, especially the training of therapists might be further improved.

Therapist training in the current study contained an overview of MI and all of its aspects as well as training of different techniques. From the feedback of the trained therapists, it might be of benefit to keep the overview part to a minimum in favor of focusing on the core techniques to be used in the intervention. The focus can thereby be put on the training of basic MI skills such as the OARS techniques and giving information and advice. For further development of the therapist training, we would add a focus on techniques for rolling with resistance since therapists identified these as especially useful with patients with AN.

Another suggestion by therapists to the MANNA intervention was the inclusion of significant others and families. Outpatient interventions such as the MANTRA treatment (21) routinely incorporate significant others and dedicate a whole part of their treatment manual to this topic, thus emphasizing its importance. For our inpatient manual however, since sessions with significant others and relatives are an inherent part of the multidisciplinary treatment approach and not exclusive to individual psychotherapy, we did not dedicate a specific worksheet to this. It might be useful in the future however, to either offer an optional worksheet that can be used at any given time in the intervention or at least provide some information on MI and the inclusion of significant others and relatives into the treatment.

The current study contains some limitations that need to be mentioned. First of all, the sample size was small for a comparative study in this pilot phase of the evaluation of the new manual. Potentially due to the even smaller sample at the end of treatment, some of the utilized measures did not reach a satisfactory reliability at the last measurement time point. Although significant differences in e.g., dropout rates were found, this results in a wider variability and therefore a large confidence interval for this finding. The replication of these findings in a larger sample should therefore be aimed for.

Another limitation resulting from the small sample size is the lack of subgroup analyses as well as analyses of potentially confounding variables such as therapist effects that could not be investigated in the context of this study. These analyses enable tailoring the MANNA intervention to specific subgroups and help differentiate cases in which other/additional interventions are needed for example due to cognitive impairments of patients due to the severe state of malnutrition. A future, fully powered RCT on the concept will add to the evidence base through more in-depth statistical analyses (e.g., survival analysis) but also minimize risk of biases through e.g., rater votings of therapist behaviors with the Motivational Interviewing Skill Code (39) that was not possible in this pilot study setting.

In conclusion, this pilot study confirms high acceptance and very good feasibility of the newly developed MANNA intervention for the treatment of inpatients with AN. Although the sample size was relatively small and no significant differences concerning stages of change and treatment outcomes were found, patients receiving the MANNA interventions finished treatment on regular terms significantly more often than patients in the control intervention, thus pointing at potential benefits in crucial dimensions of the therapy of AN.

## Data Availability Statement

The raw data supporting the conclusions of this article will be made available by the authors, without undue reservation.

## Ethics Statement

The studies involving human participants were reviewed and approved by the ethics committee of the medical faculty of the University of Tuebingen and the ethics committee of the medical faculty of the University of Duisburg-Essen. The patients/participants provided their written informed consent to participate in this study.

## Author Contributions

KZ, KK, SB, GR, KEG, SZ, and FJ contributed to the conception and design of the study. KZ, NR, SB, E-MS, and MT substantially contributed to the acquisition of data for the study. KZ performed the statistical analysis and wrote the first draft of the manuscript. All authors contributed to manuscript revision and read and approved the submitted version.

## Conflict of Interest

The authors declare that the research was conducted in the absence of any commercial or financial relationships that could be construed as a potential conflict of interest.
